# Preterm birth: associated risk factors in the tertiary care center

**DOI:** 10.12688/f1000research.154079.1

**Published:** 2024-10-11

**Authors:** Sweety Jousline Fernandes, Tessy Treesa Jose, Judith Angelitta Noronha, Sushmitha Karkada

**Affiliations:** 1 MSc (N), MPhil(N), Asst Professor, OBG Dept., MCON, Manipal Academy of Higher Education, Manipal, Karnataka, 576104, India; 2 MSc (N), Ph.D. (N) Professor and Head, Dept of Mental health (Psychiatry) (N), MCON, Manipal Academy of Higher Education, Manipal, Karnataka, 576104, India; 3 MSc (N), MPhil. (N), Ph.D. (N), Dean, Professor MCON, Manipal Academy of Higher Education, Manipal, Karnataka, 576104, India; 4 MSc (N), Asst Professor(Sr Scale), OBG Dept., MCON, Manipal Academy of Higher Education, Manipal, Karnataka, India

**Keywords:** preterm labor, prevalence, risk factors

## Abstract

The study aimed at identifying the prevalence of preterm labor and the associated risk factors.

**Design:**

A quantitative approach using a retrospective case-control study.

**Setting:**

Tertiary care hospital of Udupi district Karnataka.

**Population or Sample:**

Women delivered in tertiary care Hospital of Udupi district, Karnataka, were the sample; among them, the cases (250) were the records of the women who had delivered before 37 weeks of gestation, and controls (500) were the records of women who delivered after 37 weeks of gestation and without any complications.

**Method:**

The study was conducted using a retrospective case-control design by reviewing the case records of women who had delivered in a tertiary care hospital.

**Main Outcome Measures:**

Women delivered in tertiary care Hospital of Udupi district, Karnataka, and their inpatient records were assessed for risk factors during the antenatal and delivery periods.

**Results:**

The study revealed that the prevalence of preterm labor was 356 (14.86%) Out of 2402 deliveries. Among them, only 250 were assessed. It was significantly correlated with age, place of residence, degree of education, occupation, marital status, gravid para, number of deliveries, type of deliveries, gap between births, blood type, and religion. Pregnant women who had been exposed or had a risk for preterm labor included those who had been diagnosed with pregnancy-induced hypertension, medication during pregnancy, history of abortion, intense physical labor, and conception dates older than 30 years.

**Conclusion:**

The preterm labor prevalence can be minimized if the modifiable risk factors are in control. Non-modifiable risk factors require keen supervision. Thus, health professionals must be alert to all modifiable and non-modifiable risk factors.

## Introduction

A neonate born after 37 weeks requires significant attention and care as they transition into a new environment post-birth. The morbidity associated with preterm labor can persist into later life, leading to physical, psychological, and economic costs. Globally, one in 10 babies is born preterm, and approximately one million babies die annually due to complications from preterm births. Preterm labor is an obstetric emergency and poses a threat to population health, contributing to 75% of infant mortality ^31^ ( https://www.who.int).

Preterm labor not only imposes financial and emotional distress on families but can also result in permanent disabilities (physical or neural) in infants. Surviving babies often exhibit periodic disabilities such as learning, visual, and hearing difficulties. The preterm birth rate ranges from 5% to 7% of live births in developed countries compared to developing countries. Despite an increased understanding of potential risk factors and their pathological mechanisms, the preterm birth rate has remained unchanged or even increased in most countries over the past two decades. ^15^^,^^16^

The pathway to preterm labor remains unclear, whether it results from the interaction of multiple pathways or an independent pathway. Factors commonly affecting preterm labor include the health condition of the mother or fetus, genetic causes, environmental exposure, infertility treatments, habits, socioeconomic factors, and iatrogenic factors. Preterm birth was the second leading cause of death in children under five years old in 2010. Of the approximately 3.1 million newborn deaths that year, a quarter occurred within the first 24 hours after birth ^14^American College of Obstetricians and Gynecologists (2020).

In India, out of 27 million neonates born each year, 3.5 million are delivered prematurely. The antenatal period, labor process, and postnatal period are critical for infant and maternal survival. Preterm labor is unpredictable, but cues can be identified, and preventive measures can be taken. Physicians strive to delay delivery to allow the baby to grow as much as possible. Therefore, pregnant women should not omit any essential health details during regular visits to the obstetrics clinic. They should provide a detailed history of their lifestyle, past pregnancies, and any health issues they have experienced and clarify any doubts—Centers for Disease Control and Prevention (2019). ^4^

The statistics presented above and the supporting data assist obstetric care providers in designing appropriate studies and planning measures to decrease delivery rates before 37 weeks of gestation and improve the health status of women who have delivered. This will help reduce and fill the gaps between study areas, serving as baseline information for other countries. The present study was conducted to identify preventive measures to determine the risk factors of preterm labor. Early identification and extra precautions for risk factors, such as medication intake during pregnancy and previous abortion, can prevent preterm labor. Moderate risk factors like hard physical work and conception age above 30 years can prevent preterm labor if managed.

**Table 1.** Frequency & percentage distribution of sample characteristics among cases & controls.

	N=750			
Sample characteristics	Case (250)		Control (500)	
	(f)	(%)	(f)	(%)
**Residence**				
Urban	59	23.6	166	33.2
Rural	191	76.4	334	66.8
**Education level**				
Illiterate	6	2.4	42	8.4
Primary/secondary school	5	2	-	-
PUC	6	2.4	125	25
Graduation	30	12	41	8.2
Not applicable	203	81.2	292	58.4
**Occupation**				
Housewife	200	80	250	50
Peasant	14	5.6	-	-
Government	6	2.4	42	8.4
Others	30	12	208	41.6
**Gravida**				
Primi	189	75.6	167	33.4
Second	38	15.2	251	50.2
Third	11	4.4	-	-
Fourth & above	12	4.8	82	16.4
**Number of deliveries**				
One	189	75.6	167	33.4
Two	61	24.4	333	66.6
**Type of delivery**				
Normal	25	10	500	100
Cesarean section	225	90	-	-
**Birth interval (in yrs.)**				
1–2	32	8.8	292	58.4
3–4	21	8.4	83	16.6
5 & above	18	7.2	-	-
Not specified	179	75.6	125	25
**Drugs received during pregnancy**				
Folic acid	250	100	500	100
Calcium	250	100	500	100
Iron	250	100	500	100
**Blood group**				
A+	59	23.6	209	41.8
B+	57	22.8	83	16.6
AB+	12	4.8	-	-
O+	111	44.4	167	33.4
A-	6	2.4	-	-
O-	5	2	41	8.2
**Religion**				
Hindu	227	90.8	375	75
Christian	23	9.2	-	-
Muslim	-	-	125	25

The data presented in the Table 1 show that majority of the pregnant women from both cases and control groups were residing in rural area 191(76.4%) and 334 (66.8%) respectively, most of the women’s educational status is not mentioned as, many of the women in both the groups were housewives (case 200 (80%) and controls 250 (50%)). All the women in both the groups were married and were with their spouses. Majority of the women among cases were primi 189 (75.6%) and majority of them 251 (50.2%) of pregnant women among control were pregnant for the second time. Majority of women among cases had delivered once 189 (75.6%) and maximum among the controls delivered twice 333(66.6%). Among the cases majority were primi so the birth interval was not specified and among the controls majority 292(58.4%) of them had birth interval of 1 – 2yrs. Women in both the group had regular follow up and had taken supplements as prescribed. Additional information on Blood grouping and religion was collected and majority women among the cases were O+ 111(44.4%) blood group and among the controls majority were A+ 209(41.8%) blood group. Among both the group, cases and control were belonging to the Hindu religion i.e. 227 (90.8%) and 375 (75%) respectively.

**Table 2.** Mean and standard deviation of continuous demographic variable among cases and controls.

	N=750			
	Cases (250)		Controls (500)	
Variables	Mean	SD	Mean	SD
**Age (years)**	27.44	3.564	27.07	3.498
**Height (in cms)**	154.32	5.918	154.34	5.695
**Total weight gain during pregnancy (in kg)**	7.67	3.815	7.54	3.746

The data presented in Table 2 show that the mean age of cases (27.44) and controls (27.07) is the same. The mean height for the cases (154.32 cm) and control group is (154.34 cm) was also the same. The total weight gain of pregnant women during the pregnancy on an average is 7.67 kgs and that of control is 7.54 kgs. There was no difference between the cases and controls.

**Table 3.** Association between the demographic variable and preterm labor.

	N=250						
Sample characteristics	Case		Control		χ ^2^	Df	P
	(f)	(%)	(f)	(%)			
**Age in years**							
19–22	15	6	42	8.4			
23–26	82	32.8	125	25	4.114	16	*.001
27–30	99	39.6	209	41.8			
31–34	49	19.6	124	24.8			
35 <	5	2	-	-			
**Residence**							
Urban	59	23.6	166	33.2	18.369	2	*.001
Rural	191	76.4	334	66.8			
**Education level**							
Illiterate	6	2.4	42	8.4			
Primary/secondary school	5	2	--	-			
PUC	6	2.4	125	25	.83.781	4	*.001
Graduation	30	12	41	8.2			
Not specified	203	81.2	292	58.4			
**Occupation**							
Housewife	200	80	250	50			
Peasant	14	5.6	-	-	1.084	3	*.001
Government	6	2.4	42	8.4			
Others	30	12	208	41.6			
**Marital status**							
Married	250	100	500	100			
Living with spouse	250	100	500	100	10.067	1	*.004
**Gravida**							
Primi	189	75.6	167	33.4			
Second	38	15.2	251	50.2	1.554	3	*.001
Third	11	4.4	-	-			
Fourth & above	12	4.8	82	16.4			
**Number of deliveries**							
Once	189	75.6	167	33.4	42.697	2	*.001
Twice	61	24.4	333	66.6			
**Type of delivery**							
Normal delivery	25	10	500	100	6.429	2	*.001
Caesarean section	225	90	-	-			
**Birth interval (in yrs.)**							
1–2	32	8.8	292	58.4			
3–4	21	8.4	83	16.6			
5 & above	18	7.2	-	-	2.136	3	*.001
Not specified	179	75.6	125	25			
**Blood group**							
A+	59	23.6	209	41.8			
B+	57	22.8	83	16.6	70.768	5	*.001
AB+	12	4.8	-	-			
O+	111	44.4	167	33.4			
A-	6	2.4	-				
O-	5	2	41	8.2			
**Religion**							
Hindu	227	90.8	375	75	1.137	3	*.001
Christian	23	9.2	-	-			
Muslim	-	-	125	25			

* Significance at the level of 0.05.

The data in the Table 3 shows that there is association with the age (p=.002), residence (p=.001), education level (p=.001), occupation (p=.001), marital status (p=.004), gravida (p=.001), number of deliveries (p=.001), type of delivery (p=.001), birth interval (p=.001), blood group (p=.001) and the religion (p=.001). But there is no association between Height and weight gain during pregnancy. Thus it states that there is high risk for the preterm labor at the age ranging from 27 – 30 years, if the women are from rural area, being at home with primi para.

**Table 4.** Logistic regression, adjusted and unadjusted Odds ratio, confidence interval of risk factors.

		N= 750				
		Cases	Controls	Unadjusted Odds	P	Adjusted Odds ratio
		(250)	(500)	ratio	value	(95%CI [LL,UL])
		Yes	Yes			
		n (%)	n (%)			
**Urinary tract infection**	Yes	6(2.4)	41(8.2)	.275(.115, .658)	.127	4.691(.644, 34.198)
	No *	244(97.6)	459(91.8)	1		1
**H/o any other chronic disease**	Yes	40(16)	42(8.4)	2.138(1.345,3.399)	.034	5.110(1.128,23.153)
	No *	210(84)	458(91.6)	1		1
**Pregnancy induced Hypertension**	Yes	109(43.6)	41(8.2)	8.654(5.769,12.984)	*.001	1.288(140.829,1.17)
	No *	141(56.4)	459(91.8)	1		1
**Medication intake during pregnancy**	Yes	173(69.2)	166(33.2)	1.117(.805, 1.548)		62.406(7.599,512.513)1
	No *	77(30.8)	334(66.8)	1	*.001	1
**Short cervical length**	Yes	235(94)	35(7)	.321(.181, .569)		.000(.000)
	No *	15(6)	465(93)	1	.989	1
**Hospitalization during pregnancy**	Yes	175(70)	141(28.2)	1.160(.835, 1.610)		.095(.012,.749)
	No	75(30)	359(71.8)	1	.026	1
**Premature rupture of membrane**	Yes	82(32.8)	35(7)	2.452(1.721,3.493)		2.269(.000, .749)
	No	168(67.2)	465(93)	1	.991	1
**Induced vaginal delivery**	Yes	28(11.2)	53(10.6)	.374(.241, .582)		1.228(.000)
	No	222(88.8)	447(89.4)	1	.998	1
**Still birth**	Yes	9(3.6)	40(8)	.074(.037, .149)	.998	.000(.000)
	No	241(96.4)	460(92)	1		1
**Abortion**	Yes	38(15.2)	51(10.2)	.544(.364, .811)		.007(.001,.066)
	No	212(84.8)	449(89.8)	1	*.001	1
**Diabetis mellitus**	Yes	40(16)	17(3.4)	2.132(1.339.3.395)		1.209(.000)
	No	210(84)	483(96.6)	1	.990	1
**Hard physical work**	Yes	14(5.6)	17(3.4)	.664(.355,1.24)		.021(.002,.217)
	No	236(94.4)	483(96.6)	1	*.001	1
**Conception at 30 or above**	Yes	46(18.4)	51(10.2)	.684(.468, .999)	24.837(2.648,232.965)	
	No	204(81.6)	449(89.8)	1	*.005	1
**Being obese pregnancy**	Yes	6(2.4)	17(3.4)	.275(.115,.658)		1.136(.000)
	No	244(97.6)	483(96.6)	1	.999	1

* Significant at the level of .005.

The data in the Table 4 shows that if the pregnant women had exposure to the pregnancy induced hypertension AOR 1.288(CI 140.829, 1.17), Medication intake during pregnancy AOR 62.406(CI 7.599,512.513), Abortion AOR .007(CI.001,.066), hard physical work AOR.021(CI.002,.217), Conception age 30 and above AOR 24.837(CI 2.648,232.965) has chances of having preterm labor.

After assessing the Adjusted Odds ratio the data shows that the risk factors like pregnancy induced hypertension (p=.001), medication intake (p=.001) and conception age at 30 or above (p=.005) are having chances of preterm labor which is proved significantly at the level of.005. Previous Abortion (p=.001) & hard physical work (p=.001) are proved statistically as the preventive factors but clinically it is not.

## Methods

This retrospective case-control study aimed to identify the prevalence and risk factors for preterm labor by examining the case records of women who delivered in tertiary care hospitals in the Udupi district. The cases were women who delivered before 37 weeks of gestation, and the controls were women who delivered after 37 weeks of gestation without any complications. As per the sample size calculation, a purposive sampling technique was employed to select the records of 250 out of 356 women.

The tools used included a maternal socio-demographic proforma and a structured Risk Assessment tool for preterm labor. This tool classified items into modifiable and non-modifiable risk factors, with the former further subdivided into social, economic, and environmental factors and the latter into medical, obstetrical, and fetal conditions.

The study received permission from various authorities related to the data and ethical clearance (IEC 30/2018), and it was registered with the Clinical Trial Registry of India (CTRI/2018/05/014078). Data were analyzed using SPSS version 16, employing descriptive and inferential statistics.

The study identified that the prevalence of preterm labor was 14.82% (356 out of 2402 deliveries). The background characteristics of the cases and controls varied, with the majority residing in rural areas and being homemakers. The mean height and age among the cases were 154.32cm and 27.44 years, respectively, and the average total weight gain during pregnancy was 7.67 kg.

Univariate analysis was initially computed, followed by the computation of the adjusted odds ratio to obtain an accurate result. The data showed that risk factors like pregnancy-induced Hypertension Hypertension (p=.001), medication intake (p=.001), and conception age at 30 or above (p=.005) are associated with preterm labor, which is significant at the 0.005 level. Previous abortion (p=.001) and hard physical work (p=.001) are statistically preventive factors but are not clinically preventive risk factors. Most women in both the case and control groups were administered medications such as Tibolone (a steroid) and Ceftriaxone (an antibiotic). Statistically, these medications were associated with an increased likelihood of preterm labor. However, from a clinical perspective, these steroids and antibiotics are typically administered as a prophylactic measure for cases that progress into labor before 37 weeks of gestation.

## Discussion

The data from this study revealed that 14.82% (356 out of 2406) of deliveries were preterm. This finding aligns with a cross-sectional study conducted on the prevalence of preterm labor among young parturient women aged 15 – 24 years attending public hospitals in Brazil, which found a high prevalence of preterm labor (86.3% out of 2400 parturient women).

Similarly, a retrospective study in Jordan identified the existence and reasons associated with preterm delivery, showing that all the preterm deliveries were approximately 647. Most neonates were female (54.9% Vs. 45.1%), and most (75.6%) were the second child. The women who delivered preterm were predominantly between 25 and 35.

This study found associations between preterm labor and several factors, including age, residence, education level, occupation, marital status, gravida, number of deliveries, type of delivery, birth interval, blood group, and religion. However, no association was found between preterm labor and height or weight gain during pregnancy.

These findings are supported by a retrospective cross-sectional study on the prevalence of preterm labor in a Labor room, which identified risk factors for early labor such as active relationships during the previous week of labor, multiple pregnancies, small birth intervals between two conceptions, PIH, fetal anomaly, premature rupture of membrane, and Hypertension.

A case-control study by Barbara Luke et al. in the United States on the relation between occupational factors and preterm delivery among nurses found that risk factors related to preterm delivery included working hours per week, different duty timings, standing hours, noisy areas, physical stress, and work-related stress.

The results of this study and similar ones suggest that eliminating risk factors and reinforcing protective factors could help decrease the rate of preterm labor and its human and social burden. However, further studies with better design, such as cohort prospective studies with a proper follow-up period and large study population, are needed to determine these factors accurately. As the Hospital studied is a referral center for these patients, it represents the general population of the country to a great extent.

## Conclusion

The study concludes that preterm labor is commonly seen in pregnant women who are exposed to non-modifiable and modifiable risk factors. Modifiable risk factors can be avoided and thus allow the pregnancy to continue till term. Non-modifiable risk factors need to be supervised very keenly so that there is no risk to the life of the mother and the fetus. It even states that the prevalence of preterm is higher among homemakers. Thus, these women need to be released from the level of stress to which they are exposed. All in all, the study concludes that these risk factors are different for each woman, which will lead to preterm labor, but they need to be identified at the earliest and treated adequately.

More information can be found on our data guidelines page. Data is available in the OSF web application: Contributors: Sweety Jousline Fernandes Identifier: https://osf.io/wp8xu/?view_only=da380485c7c74b20befc1e58f95fe431

### Reporting guidelines

Repository: Strobe checklist for ‘Prevalence and Risk factors associated with Preterm Labour’, https://doi.org/10.17605/OSF.IO/RBHXJ

## Ethical statement: Ethical consideration

The data collection for the study commenced only after obtaining prior ethical clearance from the departments, which are as listed below:

Administrative permission from Dean Manipal College of Manipal, to conduct the study.

Permission from IRC of Manipal College of Nursing Manipal for conducting the study (IRC148/2017)

Permission from IEC of Kasturba Hospital Manipal on 10/1/2018 (IEC 30/2018)

CTRI registration (CTRI/2018/05/014078) (its not a clinical trial but during this study duration it was insisted to register under clinical trial)

Permission from the OBG Department of Kasturba Hospital Manipal was obtained for the selection and utilizing the samples Records

Permission from the Medical Superintendent of the Kasturba Hospital Manipal, was obtained to have access to the Medical Record

**Consent to participate** written consent was obtained from the hospital authority and the MRD section to view the records. Based on the hospital numbers selected every day fifteen records were issued for the study assessment.

## Author contributions

**Principal Investigator**: Mrs. Sweety J Fernandes (role: Recruitment, data collection, rater, intervention provider, concept analysis, training provider, and manuscript)

**Guide:** Dr Tessy Treesa Jose (Role: Intellectual inputs, mentoring)

**Co–guide**: Dr Judith A Noronha (Role: Intellectual, mentoring)

**Co-investigator:** Dr Sushmitha R Karkada (Role: Intellectual analysis)

## Data Availability

Data is available in the OSF web application: Contributors: Sweety Jousline Fernandes Identifier: https://osf.io/wp8xu/?view_only=da380485c7c74b20befc1e58f95fe431, https://doi.org/10.17605/OSF.IO/RBHXJ. ^10^ License: *CC-By Attribution 4.0 International*
