# Preterm birth: associated risk factors in the tertiary care center

**DOI:** 10.12688/f1000research.154079.2

**Published:** 2025-04-17

**Authors:** Sweety Jousline Fernandes, Tessy Treesa Jose, Judith Angelitta Noronha, Sushmitha Karkada

**Affiliations:** 1 MSc (N), MPhil(N), Asst Professor, OBG Dept., MCON, Manipal Academy of Higher Education, Manipal, Karnataka, 576104, India; 2 MSc (N), Ph.D. (N) Professor and Head, Dept of Mental health (Psychiatry) (N), MCON, Manipal Academy of Higher Education, Manipal, Karnataka, 576104, India; 3 MSc (N), MPhil. (N), Ph.D. (N), Dean, Professor MCON, Manipal Academy of Higher Education, Manipal, Karnataka, 576104, India; 4 MSc (N), Asst Professor(Sr Scale), OBG Dept., MCON, Manipal Academy of Higher Education, Manipal, Karnataka, India

**Keywords:** preterm labor, prevalence, risk factors

## Abstract

The study aimed at identifying the prevalence of preterm labor and the associated risk factors.

**Methods:**

A quantitative approach using a retrospective case-control study was conducted at a tertiary care hospital in Udupi district, Karnataka. The sample consisted of women who delivered in the hospital. Among them, cases (250) were records of women who had delivered before 37 weeks of gestation, and controls (500) were records of women who delivered after 37 weeks ^1^ of gestation without any complications.

**Results:**

The study revealed that the prevalence of preterm labor was 356 (14.86%) out of 2402 deliveries. Among these, only 250 were assessed. Preterm labor was significantly correlated with age, place of residence, degree of education, occupation, marital status, gravida, number of deliveries, type of deliveries, gap between births, blood type, and religion. Pregnant women exposed or at risk for preterm labor included those diagnosed with pregnancy-induced hypertension, those who took medication during pregnancy, those with a history of abortion, those engaged in intense physical labor, and those who conceived after the age of 30.

**Conclusion:**

The prevalence of preterm labor can be minimized if modifiable risk factors are controlled, while non-modifiable risk factors require keen supervision. Health professionals must be alert to all modifiable and non-modifiable risk factors.

## Introduction

A neonate born after 37 weeks requires significant attention and care as they transition into a new environment post-birth. The morbidity associated with preterm labor can persist into later life, leading to physical, psychological, and economic costs. Globally, one in 10 babies is born preterm, and approximately one million babies die annually due to complications from preterm births. ^2^^–^^6^ Preterm labor is an obstetric emergency and poses a threat to population health, contributing to 75% of infant mortality. ^5^^–^^10^

Preterm labor not only imposes financial and emotional distress on families but can also result in permanent disabilities (physical or neural) in infants. Surviving babies often exhibit periodic disabilities such as learning, visual, and hearing difficulties. The preterm birth rate ranges from 5% to 7% of live births in developed countries compared to developing countries. ^11^^,^^12^ Despite an increased understanding of potential risk factors and their pathological mechanisms, the preterm birth rate has remained unchanged or even increased in most countries over the past two decades. ^13^^,^^14^

The pathway to preterm labor remains unclear, whether it results from the interaction of multiple pathways or an independent pathway. Factors commonly affecting preterm labor include the health condition of the mother or fetus, genetic causes, environmental exposure, infertility treatments, habits, socioeconomic factors, and iatrogenic factors. Preterm birth was the second leading cause of death in children under five years old in 2010. Of the approximately 3.1 million newborn deaths that year, a quarter occurred within the first 24 hours after birth (American College of Obstetricians and Gynecologists, 2020). ^15^^,^^16^

In India, out of 27 million neonates born each year, 3.5 million are delivered prematurely. The antenatal period, labor process, and postnatal period are critical for infant and maternal survival. ^11^ Preterm labor is unpredictable, but cues can be identified, and preventive measures can be taken. Physicians strive to delay delivery to allow the baby to grow as much as possible. Therefore, pregnant women should not omit any essential health details during regular visits to the obstetrics clinic. They should provide a detailed history of their lifestyle, past pregnancies, and any health issues they have experienced and clarify any doubts. ^11^

Given the high prevalence and significant impact of preterm labor, there is a crucial need to assess various risk factors associated with it. The statistics and incidence rates indicate that preterm births contribute to substantial infant mortality and long-term morbidity. ^17^ However, there is a gap in understanding the specific modifiable and non-modifiable risk factors that contribute to preterm labor in different regions, including Udupi district. Identifying these risk factors can help develop targeted interventions to reduce preterm birth rates, improve maternal and neonatal health outcomes, and alleviate the financial and emotional burden on affected families. This research aims to fill this gap by providing comprehensive data on the risk factors specific to the study population, thereby informing healthcare strategies and policies.

The statistics presented above and the supporting data assist obstetric care providers in designing appropriate studies and planning measures to decrease delivery rates before 37 weeks of gestation and improve the health status of women who have delivered. This will help reduce and fill the gaps between study areas, serving as baseline information for other countries. The present study was conducted to identify preventive measures to determine the risk factors of preterm labor. Early identification and extra precautions for risk factors, such as medication intake during pregnancy and previous abortion, can prevent preterm labor. Moderate risk factors like hard physical work and conception age above 30 years can prevent preterm labor if managed.

**Table 1.** Frequency & percentage distribution of sample characteristics among cases & controls.

	N=750			
Sample characteristics	Case (250)		Control (500)	
	(f )	(%)	(f )	(%)
**Residence**				
Urban	59	23.6	166	33.2
Rural	191	76.4	334	66.8
**Education level**				
Illiterate	6	2.4	42	8.4
Primary/secondary school	5	2	-	-
PUC	6	2.4	125	25
Graduation	30	12	41	8.2
Not applicable	203	81.2	292	58.4
**Occupation**				
Housewife	200	80	250	50
Peasant	14	5.6	-	-
Government	6	2.4	42	8.4
Others	30	12	208	41.6
**Gravida**				
Primi	189	75.6	167	33.4
Second	38	15.2	251	50.2
Third	11	4.4	-	-
Fourth & above	12	4.8	82	16.4
**Type of delivery**				
Normal	25	10	500	100
Cesarean section	225	90	-	-
**Birth interval (in yrs.)**				
1–2	32	8.8	292	58.4
3–4	21	8.4	83	16.6
5 & above	18	7.2	-	-
Not specified	179	75.6	125	25
**Drugs received during pregnancy**				
Folic acid	250	100	500	100
Calcium	250	100	500	100
Iron	250	100	500	100
**Blood group**				
A+	59	23.6	209	41.8
B+	57	22.8	83	16.6
AB+	12	4.8	-	-
O+	111	44.4	167	33.4
A-	6	2.4	-	-
O-	5	2	41	8.2
**Religion**				
Hindu	227	90.8	375	75
Christian	23	9.2	-	-
Muslim	-	-	125	25

The data presented in the Table 1 show that majority of the pregnant women from both cases and control groups were residing in rural area 191(76.4%) and 334 (66.8%) respectively, most of the women’s educational status is not mentioned as, many of the women in both the groups were housewives (case 200 (80%) and controls 250 (50%)). All the women in both the groups were married and were with their spouses. Majority of the women among cases were primi 189 (75.6%) and majority of them 251 (50.2%) of pregnant women among control were pregnant for the second time. Majority of women among cases had delivered once 189 (75.6%) and maximum among the controls delivered twice 333(66.6%). Among the cases majority were primi so the birth interval was not specified and among the controls majority 292(58.4%) of them had birth interval of 1 – 2yrs. Women in both the group had regular follow up and had taken supplements as prescribed. Additional information on Blood grouping and religion was collected and majority women among the cases were O+ 111(44.4%) blood group and among the controls majority were A+ 209(41.8%) blood group. Among both the group, cases and control were belonging to the Hindu religion i.e. 227 (90.8%) and 375 (75%) respectively.

**Table 2.** Mean and standard deviation of continuous demographic variable among cases and controls.

	N=750			
	Cases (250)		Controls (500)	
Variables	Mean	SD	Mean	SD
**Age (years)**	27.44	3.564	27.07	3.498
**Height (in cms)**	154.32	5.918	154.34	5.695
**Total weight gain during pregnancy (in kg)**	7.67	3.815	7.54	3.746

The data presented in Table 2 show that the mean age of cases (27.44) and controls (27.07) is the same. The mean height for the cases (154.32 cm) and control group is (154.34 cm) was also the same. The total weight gain of pregnant women during the pregnancy on an average is 7.67 kgs and that of control is 7.54 kgs. There was no difference between the cases and controls.

**Table 3.** Association between the demographic variable and preterm labor.

	N=250						
Sample characteristics	Case		Control		χ ^2^	Df	P
	(f )	(%)	(f )	(%)			
**Age in years**							
19–22	15	6	42	8.4			
23–26	82	32.8	125	25	4.114	16	*.001
27–30	99	39.6	209	41.8			
31–34	49	19.6	124	24.8			
35 <	5	2	-	-			
**Residence**							
Urban	59	23.6	166	33.2	18.369	2	*.001
Rural	191	76.4	334	66.8			
**Education level**							
Illiterate	6	2.4	42	8.4			
Primary/secondary school	5	2	--	-			
PUC	6	2.4	125	25	.83.781	4	*.001
Graduation	30	12	41	8.2			
Not specified	203	81.2	292	58.4			
**Occupation**							
Housewife	200	80	250	50			
Peasant	14	5.6	-	-	1.084	3	*.001
Government	6	2.4	42	8.4			
Others	30	12	208	41.6			
**Marital status**							
Married	250	100	500	100			
Living with spouse	250	100	500	100	10.067	1	*.004
**Gravida**							
Primi	189	75.6	167	33.4			
Second	38	15.2	251	50.2	1.554	3	*.001
Third	11	4.4	-	-			
Fourth & above	12	4.8	82	16.4			
**Number of deliveries**							
Once	189	75.6	167	33.4	42.697	2	*.001
Twice	61	24.4	333	66.6			
**Type of delivery**							
Normal delivery	25	10	500	100	6.429	2	*.001
Caesarean section	225	90	-	-			
**Birth interval (in yrs.)**							
1–2	32	8.8	292	58.4			
3–4	21	8.4	83	16.6			
5 & above	18	7.2	-	-	2.136	3	*.001
Not specified	179	75.6	125	25			
**Blood group**							
A+	59	23.6	209	41.8			
B+	57	22.8	83	16.6	70.768	5	*.001
AB+	12	4.8	-	-			
O+	111	44.4	167	33.4			
A-	6	2.4	-				
O-	5	2	41	8.2			
**Religion**							
Hindu	227	90.8	375	75	1.137	3	*.001
Christian	23	9.2	-	-			
Muslim	-	-	125	25			

* Significance at the level of 0.05.

The data in the Table 3 shows that there is association with the age (p=.002), residence (p=.001), education level (p=.001), occupation (p=.001), marital status (p=.004), gravida (p=.001), number of deliveries (p=.001), type of delivery (p=.001), birth interval (p=.001), blood group (p=.001) and the religion (p=.001). But there is no association between Height and weight gain during pregnancy. Thus it states that there is high risk for the preterm labor at the age ranging from 27 – 30 years, if the women are from rural area, being at home with primi para.

**Table 4.** Logistic regression, adjusted and unadjusted Odds ratio, confidence interval of risk factors.

		N= 750				
		Cases	Controls	Unadjusted Odds	P	Adjusted Odds ratio
		(250)	(500)	ratio	value	(95%CI [LL,UL])
		Yes	Yes			
		n (%)	n (%)			
**Urinary tract infection**	Yes	6(2.4)	41(8.2)	.275(.115, .658)	.127	4.691(.644, 34.198)
	No *	244(97.6)	459(91.8)	1		1
**H/o any other chronic disease**	Yes	40(16)	42(8.4)	2.138(1.345,3.399)	.034	5.110(1.128,23.153)
	No *	210(84)	458(91.6)	1		1
**Pregnancy induced Hypertension**	Yes	109(43.6)	41(8.2)	8.654(5.769,12.984)	*.001	1.288(140.829,1.17)
	No *	141(56.4)	459(91.8)	1		1
**Medication intake during pregnancy**	Yes	173(69.2)	166(33.2)	1.117(.805, 1.548)		62.406(7.599,512.513)1
	No *	77(30.8)	334(66.8)	1	*.001	1
**Short cervical length**	Yes	235(94)	35(7)	.321(.181, .569)		.000(.000)
	No *	15(6)	465(93)	1	.989	1
**Hospitalization during pregnancy**	Yes	175(70)	141(28.2)	1.160(.835, 1.610)		.095(.012,.749)
	No	75(30)	359(71.8)	1	.026	1
**Premature rupture of membrane**	Yes	82(32.8)	35(7)	2.452(1.721,3.493)		2.269(.000, .749)
	No	168(67.2)	465(93)	1	.991	1
**Induced vaginal delivery**	Yes	28(11.2)	53(10.6)	.374(.241, .582)		1.228(.000)
	No	222(88.8)	447(89.4)	1	.998	1
**Still birth**	Yes	9(3.6)	40(8)	.074(.037, .149)	.998	.000(.000)
	No	241(96.4)	460(92)	1		1
**Abortion**	Yes	38(15.2)	51(10.2)	.544(.364, .811)		.007(.001,.066)
	No	212(84.8)	449(89.8)	1	*.001	1
**Diabetis mellitus**	Yes	40(16)	17(3.4)	2.132(1.339.3.395)		1.209(.000)
	No	210(84)	483(96.6)	1	.990	1
**Hard physical work**	Yes	14(5.6)	17(3.4)	.664(.355,1.24)		.021(.002,.217)
	No	236(94.4)	483(96.6)	1	*.001	1
**Conception at 30 or above**	Yes	46(18.4)	51(10.2)	.684(.468, .999)	24.837(2.648,232.965)	
	No	204(81.6)	449(89.8)	1	*.005	1
**Being obese pregnancy**	Yes	6(2.4)	17(3.4)	.275(.115,.658)		1.136(.000)
	No	244(97.6)	483(96.6)	1	.999	1

* Significant at the level of .005.

The data in the Table 4 shows that if the pregnant women had exposure to the pregnancy induced hypertension AOR 1.288(CI 140.829, 1.17), Medication intake during pregnancy AOR 62.406(CI 7.599,512.513), Abortion AOR .007(CI.001,.066), hard physical work AOR.021(CI.002,.217), Conception age 30 and above AOR 24.837(CI 2.648,232.965) has chances of having preterm labor.

After assessing the Adjusted Odds ratio the data shows that the risk factors like pregnancy induced hypertension (p=.001), medication intake (p=.001) and conception age at 30 or above (p=.005) are having chances of preterm labor which is proved significantly at the level of.005. Previous Abortion (p=.001) & hard physical work (p=.001) are proved statistically as the preventive factors but clinically it is not.

Confounders are selected based on their potential to influence both the exposure and the outcome. In this table:

• Confounders such as age, previous medical history, socio-economic status, etc., might have been adjusted based on their relevance to the risk factors being studied.
• Reasons for Inclusion: The goal is to control for variables that could distort the true relationship between the risk factors and the outcomes being analyzed. By adjusting for these confounders, the analysis aims to isolate the effect of the specific risk factors on the outcomes more accurately.

## Methods

This retrospective case-control study aimed to identify the prevalence and risk factors for preterm labor by examining the case records of women who delivered in tertiary care hospitals in the Udupi district. The cases were women who delivered before 37 weeks of gestation, and the controls were women who delivered after 37 weeks of gestation without any complications. As per the sample size calculation, a purposive sampling technique was employed to select the records of 250 out of 356 women.

**Study Timeline and Record Selection**

• Timelines: The records reviewed spanned from January 2016 to December 2016.
• Criteria for Record Inclusion: Records were included if they met the criteria for gestational age determination based on the last menstrual period (LMP) or early ultrasound.
• Exclusion of Records: Records with missing data were excluded from the study, resulting in the exclusion of 50 records.
• A purposive sampling technique was employed to select the records, resulting in a sample size of 250 cases (preterm deliveries) and 500 controls (full-term deliveries), maintaining a case-to-control ratio of 1:2.
• Total Records Reviewed: 2456
•   Excluded Records: 50 (due to missing data)
•    Included Records: 2406
•     Cases (preterm deliveries before 37 weeks): 250
•     Controls (full-term deliveries after 37 weeks): 500

**Sample Selection**

• **Sample Size Calculation:** The sample size for cases (250) was purposively selected from the 356 identified preterm deliveries.
• **Cases to Controls Ratio:** A 1:2 ratio was used for case-control selection, resulting in 500 control records (full-term deliveries without complications).

The tools used included a maternal socio-demographic proforma and a structured Risk Assessment tool for preterm labor. This tool classified items into modifiable and non-modifiable risk factors, with the former further subdivided into social, economic, and environmental factors and the latter into medical, obstetrical, and fetal conditions.

**The study utilized a structured Risk Assessment tool**

• **Tool Development:** The tool was prepared by the researchers and validated through expert review and pilot testing.
• **Classification of Risk Factors:** The tool categorized risk factors into modifiable (social, economic, and environmental) and non-modifiable (medical, obstetrical, and fetal) factors.
• The tool was prepared by the researchers and validated through expert review and pilot testing. It was also validated by assessing its reliability and consistency using statistical measures such as Cronbach’s alpha. ^18^^–^^20^

**Data Analysis Plan**

• **Software Used:** Data were analyzed using SPSS version 16.
• **Descriptive Statistics:** Used to summarize the demographic and clinical characteristics of the study population.
• **Univariate Analysis:** Initially computed to identify potential risk factors.
• **Multivariate Logistic Regression:** Adjusted odds ratios were calculated to obtain more accurate results, considering multiple variables simultaneously.

The study received permission from various authorities related to the data and ethical clearance (IEC 30/2018), and it was registered with the Clinical Trial Registry of India (CTRI/2018/05/014078). Data were analyzed using SPSS version 16, employing descriptive and inferential statistics. ^21^

The study identified that the prevalence of preterm labor was 14.82% (356 out of 2402 deliveries). The background characteristics of the cases and controls varied, with the majority residing in rural areas and being homemakers. The mean height and age among the cases were 154.32 cm and 27.44 years, respectively, and the average total weight gain during pregnancy was 7.67 kg.

Religion was considered as a factor influencing preterm birth because cultural beliefs and practices, including religious ones, can significantly impact health behaviors and outcomes. For instance, religious beliefs may influence decisions about seeking medical care, adherence to prenatal care recommendations, and the use of traditional medicines or rituals1.

Univariate analysis was initially computed, followed by the computation of the adjusted odds ratio to obtain an accurate result. The data showed that risk factors like pregnancy-induced Hypertension Hypertension (p=.001), medication intake (p=.001), and conception age at 30 or above (p=.005) are associated with preterm labor, which is significant at the 0.005 level. Previous abortion (p=.001) and hard physical work (p=.001) are statistically preventive factors but are not clinically preventive risk factors. Most women in both the case and control groups were administered medications such as Tibolone (a steroid) and Ceftriaxone (an antibiotic). Statistically, these medications were associated with an increased likelihood of preterm labor. However, from a clinical perspective, these steroids and antibiotics are typically administered as a prophylactic measure for cases that progress into labor before 37 weeks of gestation. ^22^^,^^23^

### Clarification on medication statements

The statement that medications were associated with an increased likelihood of preterm labor reflects their use in clinical practice. These medications are often given to women already at risk of preterm labor to mitigate complications. Therefore, their use is more common among women who experience preterm labor, creating a statistical association.

### Corrected understanding

Steroids and antibiotics should not be seen as risk factors for causing preterm labor. Instead, they are important preventive measures administered to manage the risks associated with preterm labor and improve outcomes for the baby.

The study revealed a significant prevalence of preterm labor at 14.82% among deliveries in the tertiary care hospital of Udupi district. The findings highlight key risk factors associated with preterm labor, including pregnancy-induced hypertension, medication intake, and conception at or above age 30. While previous abortion and hard physical work appeared as statistically preventive factors, they were not clinically significant. The use of prophylactic medications like Tibolone and Ceftriaxone was noted to increase the likelihood of preterm labor statistically, emphasizing the need for careful consideration in clinical settings.

These results underscore the importance of identifying and managing risk factors to minimize preterm births. Targeted interventions and vigilant prenatal care can help mitigate the physical, psychological, and economic burdens associated with preterm labor, ultimately improving maternal and neonatal health outcomes in the region. Further research with larger populations and longitudinal studies is recommended to refine the understanding of these risk factors and enhance preventive strategies.

## Discussion

The data from this study revealed that 14.82% (356 out of 2406) of deliveries were preterm. This finding aligns with a cross-sectional study conducted on the prevalence of preterm labor among young parturient women aged 15 – 24 years attending public hospitals in Brazil, which found a high prevalence of preterm labor (86.3% out of 2400 parturient women). ^5^^,^^6^^,^^11^^,^^24^

Similarly, a retrospective study in Jordan identified the existence and reasons associated with preterm delivery, showing that all the preterm deliveries were approximately 647. Most neonates were female (54.9% Vs. 45.1%), and most (75.6%) were the second child. The women who delivered preterm were predominantly between 25 and 35. ^4^^,^^24^^–^^26^

This study found associations between preterm labor and several factors, including age, residence, education level, occupation, marital status, gravida, number of deliveries, type of delivery, birth interval, blood group, and religion. However, no association was found between preterm labor and height or weight gain during pregnancy.

These findings are supported by a retrospective cross-sectional study on the prevalence of preterm labor in a Labor room, which identified risk factors for early labor such as active relationships during the previous week of labor, multiple pregnancies, small birth intervals between two conceptions, PIH, fetal anomaly, premature rupture of membrane, and Hypertension. ^8^^,^^13^^,^^27^

A case-control study by Barbara Luke et al. in the United States on the relation between occupational factors and preterm delivery among nurses found that risk factors related to preterm delivery included working hours per week, different duty timings, standing hours, noisy areas, physical stress, and work-related stress (American College of Obstetricians and Gynecologists, 2020). ^7^^,^^28^^–^^30^

The results of this study and similar ones suggest that eliminating risk factors and reinforcing protective factors could help decrease the rate of preterm labor and its human and social burden. ^31^^,^^32^ However, further studies with better design, such as cohort prospective studies with a proper follow-up period and large study population, are needed to determine these factors accurately. As the Hospital studied is a referral center for these patients, it represents the general population of the country to a great extent. ^11^

The study identified a significant prevalence of preterm labor at 14.82% among deliveries in the tertiary care hospital of Udupi district. Key risk factors such as pregnancy-induced hypertension, medication intake, and conception at or above age 30 were significantly associated with preterm labor. The study’s findings align with other international studies, highlighting the global relevance of understanding and addressing these risk factors. ^33^^,^^34^

**Limitations of the Study**

The cross-sectional and retrospective design limits the ability to establish causality. ^19^ Conducted in a single tertiary care hospital, the findings may not be fully representative of the broader population, affecting generalizability. Recall bias from participants’ self-reported data can lead to inaccuracies. While several confounders were adjusted for, unmeasured factors might still influence the results. Measurement imprecision, particularly with gestational age, and potential selection bias if the sample is not representative are notable concerns. Loss to follow-up or missing data could also affect the robustness of the findings.

**Generalisability of the Study Results**

The findings from this study, conducted in a tertiary care hospital in Udupi district, may not be fully representative of other populations or settings due to several factors:

1. **Population Characteristics:** The specific demographics and healthcare practices in Udupi may differ from those in other regions, limiting broader applicability.
2. **Healthcare Setting:** The study’s single-center design may not reflect the diversity of other healthcare facilities.
3. **Cultural and Regional Differences:** Unique cultural, social, and environmental factors in the study location might affect generalizability.
4. **Sample Size:** A larger and more diverse population is needed to validate these findings.
5. **Study Design:** The cross-sectional and retrospective nature of the study limits its generalizability over time. Prospective studies with larger, multi-center populations are needed for robust results.

The researchers did consider fetal factors such as Intrauterine Growth Restriction (IUGR) and abnormal presentation in their analysis. However, they did not find any significant association between these factors and preterm birth. This outcome might be due to various reasons, including sample size limitations, variations in data quality, or the complex interplay of multiple factors influencing preterm birth. It highlights the need for further research with larger and more diverse populations to better understand the role of fetal factors in preterm births.

## Conclusion

The study concludes that preterm labor is commonly seen in pregnant women who are exposed to non-modifiable and modifiable risk factors. Modifiable risk factors can be avoided and thus allow the pregnancy to continue till term. Non-modifiable risk factors need to be supervised very keenly so that there is no risk to the life of the mother and the fetus. It even states that the prevalence of preterm is higher among homemakers. ^35^ Thus, these women need to be released from the level of stress to which they are exposed. All in all, the study concludes that these risk factors are different for each woman, which will lead to preterm labor, but they need to be identified at the earliest and treated adequately.

More information can be found on our data guidelines page. Data is available in the OSF web application: Contributors: Sweety Jousline Fernandes Identifier: https://osf.io/wp8xu/?view_only=da380485c7c74b20befc1e58f95fe431

### Reporting guidelines

Repository: Strobe checklist for ‘Prevalence and Risk factors associated with Preterm Labour’, https://doi.org/10.17605/OSF.IO/RBHXJ

## Ethical statement: Ethical consideration

The data collection for the study commenced only after obtaining prior ethical clearance from the departments, which are as listed below:

Administrative permission from Dean Manipal College of Manipal, to conduct the study.

Permission from IRC of Manipal College of Nursing Manipal for conducting the study (IRC148/2017)

Permission from IEC of Kasturba Hospital Manipal on 10/1/2018 (IEC 30/2018)

CTRI registration (CTRI/2018/05/014078) (its not a clinical trial but during this study duration it was insisted to register under clinical trial)

Permission from the OBG Department of Kasturba Hospital Manipal was obtained for the selection and utilizing the samples Records

Permission from the Medical Superintendent of the Kasturba Hospital Manipal, was obtained to have access to the Medical Record

**Consent to participate** written consent was obtained from the hospital authority and the MRD section to view the records. Based on the hospital numbers selected every day fifteen records were issued for the study assessment.

## Author contributions

**Principal Investigator**: Mrs. Sweety J Fernandes (role: Recruitment, data collection, rater, intervention provider, concept analysis, training provider, and manuscript)

**Guide:** Dr Tessy Treesa Jose (Role: Intellectual inputs, mentoring)

**Co–guide**: Dr Judith A Noronha (Role: Intellectual, mentoring)

**Co-investigator:** Dr Sushmitha R Karkada (Role: Intellectual analysis)

## Data Availability

Data is available in the OSF web application: Contributors: Sweety Jousline Fernandes Identifier: https://osf.io/wp8xu/?view_only=da380485c7c74b20befc1e58f95fe431, https://doi.org/10.17605/OSF.IO/RBHXJ. ^21^ License: *CC-By Attribution 4.0 International*
