# Supplementary material for: Preterm birth: associated risk factors in the tertiary care center
Source: F1000Res. 2026 Mar 18;13:1213. Originally published 2024 Oct 11. [Version 4] doi: 10.12688/f1000research.154079.4 (PMC12756588; doi:10.12688/f1000research.154079.4)
Supplement: Peer review history

# Reviewer report: Reviewer response for version 3

**DOI:** 10.5256/f1000research.187392.r439804

**Published:** 2025-12-31

**Authors:** Suriya Kumareswaran

**Affiliations:** 1 Johor State Health Department, Johor Bahru, Malaysia

The manuscript is generally clear and well-organized, but there are some recurring terminological inconsistencies—specifically, the alternating use of “preterm labor” and “preterm birth,” which may confuse readers. It would strengthen the clarity of the paper to adopt a consistent term throughout. While the authors have cited a range of references, the discussion section still lacks sufficient engagement with recent international literature, especially from low-resource settings. Incorporating comparisons with similar studies from regions like Sub-Saharan Africa or Southeast Asia would improve the contextual relevance of the findings. In several areas, citations appear clustered or not directly linked to specific arguments, which reduces their impact.

 The study design—a retrospective case-control format—is broadly appropriate for identifying associations between risk factors and preterm birth. The 1:2 ratio of cases to controls is methodologically sound. However, the transparency of the record selection process is limited by the absence of a flow diagram (as recommended by STROBE guidelines). Although inclusion and exclusion criteria are mentioned, further clarity is needed on how gestational age was verified, especially in cases with discrepancies between last menstrual period (LMP) and early ultrasound. These methodological details are essential for reproducibility and should be expanded upon.

 Regarding reproducibility, while the authors mention that a structured Risk Assessment Tool was developed and validated, no data are provided on the tool's psychometric properties, such as validity coefficients or Cronbach’s alpha. This limits confidence in the tool’s robustness. Additionally, the variables adjusted for in the multivariate logistic regression are not explicitly listed, and it’s unclear whether a proper univariate-to-multivariate transition was followed. Although the study references an OSF repository, access to raw datasets, summary tables, or a comprehensive data dictionary is not provided in the article, reducing its reproducibility.

 The statistical analysis, while conceptually appropriate, includes some issues that need to be addressed. For example, the adjusted odds ratio (AOR) for medication intake is reported as 62.4, which is implausibly high and likely reflects confounding by indication—where the medication itself is not the cause of preterm birth, but rather an indicator of high-risk pregnancies. Similarly, some confidence intervals are extremely wide (e.g., conception age ≥30 years: 2.6–232.9), suggesting low statistical precision and possible model instability. The term “statistically preventive but not clinically preventive” is used but is unclear from an epidemiological perspective and could mislead readers. It would be better to simply explain such findings as possibly due to residual confounding or reverse causality.

 The conclusions drawn are mostly supported by the findings, but the discussion would benefit from greater attention to limitations. For instance, while the study does acknowledge the limits of generalizability due to its single-center design, it overlooks important fetal factors (such as intrauterine growth restriction or congenital anomalies) which are known contributors to preterm birth. The omission of such variables, likely due to incomplete data, should be explicitly acknowledged in the limitations section. Additionally, the conclusions should be reworded to avoid overstating the general applicability of the results.

 That said, the study has notable strengths. It is conducted in a high-burden, underrepresented region (Udupi district), and involves a relatively large sample size (n=750). The authors made commendable efforts to include both modifiable and non-modifiable risk factors in their analysis, and the research was conducted ethically, with IRB and CTRI approvals in place.

 To improve the scientific rigor of this paper, the authors should: (1) add a clear flowchart showing record selection; (2) explicitly list the confounders included in the multivariate model and justify their inclusion; (3) clarify that the association of medications with preterm birth is likely due to confounding by indication rather than causation; (4) expand the discussion of limitations, especially regarding wide confidence intervals, lack of fetal data, and generalizability; and (5) strengthen the international comparison of results by incorporating relevant global literature.

**Overall, I recommend acceptance with major revisions.** The topic is highly relevant, and the dataset is valuable, but the manuscript would benefit from improvements in methodological transparency, statistical interpretation, and contextual discussion.

Is the work clearly and accurately presented and does it cite the current literature?

Partly

If applicable, is the statistical analysis and its interpretation appropriate?

Partly

Are all the source data underlying the results available to ensure full reproducibility?

Partly

Is the study design appropriate and is the work technically sound?

Partly

Are the conclusions drawn adequately supported by the results?

Partly

Are sufficient details of methods and analysis provided to allow replication by others?

Partly

Reviewer Expertise:

Maternal and child health, reproductive epidemiology, nursing research methodology, women’s health risk factors

I confirm that I have read this submission and believe that I have an appropriate level of expertise to confirm that it is of an acceptable scientific standard, however I have significant reservations, as outlined above.

# Reviewer report: Reviewer response for version 2

**DOI:** 10.5256/f1000research.176430.r387703

**Published:** 2025-07-26

**Authors:** Elvie Zulka K Rachmawati

**Affiliations:** 1 Universitas Indonesia, Depok, West Java, Indonesia

**Is the work clearly and accurately presented and does it cite the current literature?**

**Partly** – improved citations, still needs more in discussion for global context.

**Is the study design appropriate and is the work technically sound?**

**Partly** – generally reasonable retrospective design but method reproducibility incomplete.

**Are sufficient details of methods and analysis provided to allow replication by others?**

**Partly** – still missing explicit multivariable model confounder list, flowchart for included/excluded records.

**Is the statistical analysis and its interpretation appropriate?**

**Partly** – performed but overinterpreted, needs clearer caution on wide CIs and possible reverse causation.

**Are all the source data underlying the results available to ensure full reproducibility?**

**Partly** – dataset in OSF is claimed but not fully transparent (no direct dataset summary presented in paper).

**Are the conclusions drawn adequately supported by the results?**

**Partly** – conclusions generally align, but certain findings (e.g. very high AORs) overemphasized without appropriate caution.

 ///

 This manuscript by Fernandes et al. addresses the prevalence and risk factors associated with preterm birth in a tertiary care center in Karnataka, India, using a retrospective case-control design. The study investigates 250 cases of preterm deliveries against 500 controls.

**Major points needing attention:**

1. **Statistical Analysis:**
  - The statistical interpretation is partly appropriate. While univariate and multivariate analyses were performed, adjusted odds ratios are inconsistently interpreted. For instance, an AOR of 62.4 for medication intake is implausibly high and requires discussion on possible confounding by indication (i.e., medication given because of preterm risk rather than causing it).
  - Confidence intervals reported in several tables are excessively wide (e.g., conception age ≥30 years AOR 24.8 [2.6–232.9]), reflecting limited power or model instability, which warrants explicit caution in interpretation.
2. **Methodological detail & reproducibility:**
  - The retrospective design is acknowledged, but timelines and selection details, while improved from the first version, still lack a detailed flow diagram (STROBE recommended).
  - Criteria for gestational age determination were only briefly mentioned (LMP/early USG) without quality control or handling of discrepancies.
  - There is no clear list of all confounders included in the multivariate model or justification for their selection. This is critical for interpreting the adjusted associations.
3. **Data availability & source data:**
  - Although the authors mention an OSF repository, there is no direct link to raw or summary tables for re-analysis, nor a clear data dictionary, limiting full reproducibility.
4. **Discussion quality:**
  - The discussion remains superficial. The concept of "statistically preventive but clinically not preventive" is confusing and requires proper epidemiological explanation.
  - The role of medications (steroids/antibiotics) is discussed inadequately—these are typically markers of imminent preterm risk, not independent causal factors. This nuance must be made clear.
  - No exploration of fetal factors (IUGR, anomalies) was performed or reported despite mentioning them in general risk factor lists. This should be acknowledged as a limitation.
5. **Generalisability:**
  - The study’s conclusions, while valid for the center, require stronger caveats about generalizability given the single-center, referral-based, hospital population and retrospective data.
6. **Presentation & literature:**
  - Some inconsistencies remain (terms “preterm labor” vs “preterm birth”).

Citations improved but are still not consistently tied into discussion paragraphs. More international comparisons could strengthen the interpretation.

Is the work clearly and accurately presented and does it cite the current literature?

Partly

If applicable, is the statistical analysis and its interpretation appropriate?

Partly

Are all the source data underlying the results available to ensure full reproducibility?

Partly

Is the study design appropriate and is the work technically sound?

Partly

Are the conclusions drawn adequately supported by the results?

Partly

Are sufficient details of methods and analysis provided to allow replication by others?

Partly

Reviewer Expertise:

My main areas of research, particularly in pediatric otolaryngology include: infant & children feeding disorders, sleep-disordered breathing, dysphagia/swallowing difficulties, laryngopharyngeal reflux, and laryngomalacia. Preterm birth is a significant factor associated with conditions that are in my areas of research;  respiratory-feeding-reflux-postural developmental issues in neonates-infant-to children.

I confirm that I have read this submission and believe that I have an appropriate level of expertise to confirm that it is of an acceptable scientific standard, however I have significant reservations, as outlined above.

# Reviewer report: Reviewer response for version 2

**DOI:** 10.5256/f1000research.176430.r378660

**Published:** 2025-05-08

**Authors:** Dr Nalini D.S.J

**Affiliations:** 1 Sri Ramachandra institute of HigherEducation and Research (Deemed to be University), Porur, TN, India

The authors have addressed the previously suggested corrections by the reviewer appropriately in a satisfying manner.

 Abstract has been revised. Method section has been strengthened. Sources cited wherever necessary.

Is the work clearly and accurately presented and does it cite the current literature?

Partly

If applicable, is the statistical analysis and its interpretation appropriate?

Partly

Are all the source data underlying the results available to ensure full reproducibility?

Partly

Is the study design appropriate and is the work technically sound?

Partly

Are the conclusions drawn adequately supported by the results?

Partly

Are sufficient details of methods and analysis provided to allow replication by others?

Partly

Reviewer Expertise:

Women's health. Neonatology, Teaching  methodologies

I confirm that I have read this submission and believe that I have an appropriate level of expertise to confirm that it is of an acceptable scientific standard.

# Reviewer report: Reviewer response for version 1

**DOI:** 10.5256/f1000research.169062.r341100

**Published:** 2024-12-04

**Authors:** Dr Nalini D.S.J

**Affiliations:** 1 Sri Ramachandra institute of HigherEducation and Research (Deemed to be University), Porur, TN, India

I appreciate the authors for taking efforts to conduct and present the study results. The STROBE guidelines has been used for reporting the results. Wish to recommend these points for consideration.  Consistency in the term used Preterm birth or Preterm labour. In the key words only preterm labour is mentioned and not preterm birth.

 In the abstract, the concepts of design, setting, population, sample, etc., can be placed under the main heading of methods. Recent statistics of preterm birth  can be provided.

 Follow the style of citation suggested by the journal. The citation numbers are not sequential. The discussion part does not project any citations.  The introduction projects about the preterm statistics. With many published studies existing on factors associated with preterm birth, the researchers can justify the gap that their study tries to fill. The lines projected to justify the need for early identification in Page1 seems to be like assumption predicted after viewing the study results. They can be general statements on modifiable / non modifiable risk factors  or reviews from other studies be added with citation.  

 The methods section needs to be strengthened. In retrospective studies, it is important to mention the timelines (dates/ months) of reviewed records, the criteria set for considering the record for the study, number of records excluded with missed data and also project a flowchart for inclusions of record in the study. Include the criteria for gestational age determination.  On what basis was the samples for cases and control determined, the ratio used for cases: controls. Was the tool used a standardized one or was it prepared by researcher and further validated? . The plan for data analysis has to be detailed.

 In the results part, in table 1,the gravidae of women can be categorized as prime / multi or as one, two, three, etc.. Hence, a combination of both can be avoided. The researchers mention that the women had a regular follow up and took the supplements as prescribed. Being retrospective, we cannot establish whether the prescribed drugs were actually taken by subjects. Why was religion considered as a factor influencing preterm birth? Is there any supportive evidence existing? The concept of clinically preventive and statistically preventive risk factors is not clear. Steroids coverage for preterm labour cannot be ideally considered as a risk factor for PTL. The statement that medications had an increased likelihood of resulting in preterm labour seems to be contradictory. Analysis shows unadjusted estimates and, confounder-adjusted estimates and their precision (eg, 95% confidence interval). Provide clarity on which confounders were adjusted for and why they were included.

 The discussion part looks superficial and weak. In the discussion part, while projecting the prevalence of preterm births from existing literature, percentage can be used than numbers for better understanding. The potential confounders and their effects have to be discussed. Discuss the limitations of the study, taking into account sources of potential bias or imprecision. Discuss the generalisability of the study results too. Why the researchers did not consider any fetal factors namely, IUGR or abnormal presentation as a risk factor for preterm birth.

Is the work clearly and accurately presented and does it cite the current literature?

Partly

If applicable, is the statistical analysis and its interpretation appropriate?

Partly

Are all the source data underlying the results available to ensure full reproducibility?

Partly

Is the study design appropriate and is the work technically sound?

Partly

Are the conclusions drawn adequately supported by the results?

Partly

Are sufficient details of methods and analysis provided to allow replication by others?

Partly

Reviewer Expertise:

Women's health. Neonatology, Teaching  methodologies

I confirm that I have read this submission and believe that I have an appropriate level of expertise to confirm that it is of an acceptable scientific standard, however I have significant reservations, as outlined above.
